# Controlled trial of the impact of a BC adult mental health practice support program (AMHPSP) on primary health care professionals’ management of depression

**DOI:** 10.1186/s12875-018-0862-y

**Published:** 2018-11-28

**Authors:** Bianca Lauria-Horner, Tara Beaulieu, Stephanie Knaak, Rivian Weinerman, Helen Campbell, Scott Patten

**Affiliations:** 1 Department of Psychiatry, Dalhousie University, Halifax, Nova Scotia Canada; 2 Opening Minds Anti-Stigma Initiative, Mental Health Commission of Canada, Ottawa, ON Canada; 3 University of Bristish Columbia, Medical Staff Honorary Status Island Health Authority, Victoria, Canada; 4 Department of Psychiatry, University of British Columbia, Vancouver, Canada; 5 University of Calgary, Calgary, Alberta Canada

**Keywords:** Primary care, Skill-based training, Depressive disorders, Mental disorders, Patient-centered outcomes

## Abstract

**Background:**

Depression affects over 400 million people globally. The majority are seen in primary care. Barriers in providing adequate care are not solely related to physicians’ knowledge/skills deficits, but also time constraints, lack of confidence/avoidance, which need to be addressed in mental health-care redesign. We hypothesized that family physician (FP) training in the Adult Mental Health Practice Support Program (AMHPSP) would lead to greater improvements in patient depressive symptom ratings (a priori primary outcome) compared to treatment as usual.

**Methods:**

From October 2013 to May 2015, in a controlled trial 77 FP practices were stratified on the total number of physicians/practice as well as urban/rural setting, and randomized to the British Columbia AMHPSP⎯a multi-component contact-based training to enhance FPs’ comfort/skills in treating mild-moderate depression (intervention), or no training (control) by an investigator not operationally involved in the trial. FPs with a valid license to practice in NS were eligible. FPs from both groups were asked to identify 3–4 consecutive patients > 18 years old, diagnosis of depression, Patient Health Questionnaire (PHQ-9) score ≥ 10, able to read English, intact cognitive functioning. Exclusion criteria: antidepressants within 5 weeks and psychotherapy within 3 months of enrollment, and clinically judged urgent/emergent medical/psychiatric condition. Patients were assigned to the same arm as their physician. Thirty-six practices recruited patients (intervention *n* = 23; control *n* = 13). The study was prematurely terminated at 6 months of enrollment start-date due to concomitant primary health-care transformation by health-system leaders which resulted in increased in-office demands, and recruitment failure. We used the PHQ-9 to assess between-group differences at baseline, 1, 2, 3, and 6 months follow-up. Outcome collectors and assessors were blind to group assignment.

**Results:**

One hundred-and-twenty-nine patients (intervention *n* = 72; control *n* = 57) were analysed. A significant improvement in depression scores among intervention group patients emerged between 3 and 6 months, time by treatment interaction, likelihood ratio test (LR) chi2(3) = 7.96, *p* = .047.

**Conclusions:**

This novel skill-based program shows promise in translating increased FP comfort and skills managing depressed patients into improved patient clinical outcomes⎯even in absence of mental health specialists availability.

**Trial registration:**

#NCT01975948.

**Electronic supplementary material:**

The online version of this article (10.1186/s12875-018-0862-y) contains supplementary material, which is available to authorized users.

## Background

The World Health Organization (WHO) recognizes mental illness as becoming the number one cause of years-lived with disability worldwide by 2020 [1–3]. Depression is one of the most prevalent and costly conditions, affecting over 400 million people globally [4–6]. Concurrent with other physical and psychiatric conditions, there is higher morbidity and cost to the healthcare system [7]. These facts underscore the need for evidence-based strategies that promote early recognition and treatment, thereby improving patient outcomes [1, 6]. Thus, integration of mental health in primary care is ideal and has been an area of focus in mental healthcare redesign that strengthens patient-centered, evidence-based, sustainable care. Family physicians (FPs) see over 85% of these patients, and the majority can be handled early and effectively in this setting [8–15]. Most patients experience less stigma and increased comfort sharing problems with their doctor with whom they have established trust [16–18]. However, even when the diagnosis is made, less than 20% receive adequate treatment [19]. In one study conducted in 21 countries, respondents who met DSM-IV criteria for major depressive disorder (MDD) within 12 months before the interview, only 16.5% received minimally adequate treatment as defined by evidenced-based guidelines [i.e. receiving either pharmacotherapy (for a minimum of 1 month, plus 4 visits with any type of medical doctor) or psychotherapy (for a minimum of 8 visits with any professional including religious or spiritual advisor, social worker or counsellor)]. Other studies have shown that although FPs commonly prescribe antidepressants, [20] in mild/moderate cases antidepressants are not necessarily associated with improved long-term outcomes, [21–24]. Studies suggest that cognitive behaviour therapy (CBT) has an enduring effect with lower rates of relapse, that many patients prefer non-drug options, [25] and where clinically appropriate, patient choice of evidence-based options improves outcomes [26].

The extent to which training programs lead to practice changes or improved clinical outcomes remains questionable [27–31]. Barriers in providing adequate care are not solely related to physicians’ knowledge deficits, but as a result of complex interdependent factors. There are physician factors, e.g., in-office time constraints, a large number of domains to which they extend care, mental and physical health conditions being intertwined and thereby confusing symptoms; systems support factors, e.g., lack of mental health specialists, ineffective interdisciplinary teams, funding, legislation [32, 33]; and patient factors, e.g., stigma as a barrier to help-seeking, patients lack of disclosure sensing physicians’ time pressures, costly uninsured services [10, 31, 34, 35].

Training programs with top-down and bottom-up approaches such as the British Columbia (BC) Adult Mental Health Practice Support Program (AMHPSP) show the most promise as the program expands well beyond a simple education program or workshop [36, 37]. Physicians are trained to manage mild-moderate depression and anxiety disorders on their own within office time constraints by coaching patients through supported CBT-based self-management strategies. They flexibly use tools and strategies with or without antidepressants, reflecting a “real world” scenario, [38–40] and the program is based on the quality improvement framework of plan-do-study-act shown to effectively affect change [41–48] by offering FP opportunities to practice new skills immediately after the training sessions within the scope of their practice. A practice support coordinator provides on-site, in-practice support during action periods to help implement and sustain these changes [31, 49–51]. Patients are engaged through guided self-management strategies, which is key for effective chronic illness care [52–57]. Finally, physicians are compensated to attend training. In a realistic context where physicians are responsible on their own to manage these patients, or while patients wait to be seen by specialty services, we —were looking to evaluate—in a real-world environment—if training in the AMHPSP improves patient clinical outcomes. The training would provide physicians another feasible non-drug treatment option in their armamentarium.

While qualitative evaluations of the AMHPSP conducted in BC report a positive impact on several key outcomes [37], the program *has not been rigorously evaluated* through a controlled trial The NS Department of Health & Wellness and Mental Health Commission of Canada therefore sponsored a trial to evaluate its effectiveness.

The first published part of our study shows a significant improvement (diminishment) in intervention-group physicians’ preference for social distance, and significant increases in perceived confidence and comfort managing mental illness [58]. In this part of the study, we sought to determine whether training FPs in the AMHPSP would lead to greater improvements in patient depression scores (primary outcome in this part of the study) compared to the control group. This paper also focuses on patients’ satisfaction with care received, and physicians’ antidepressants prescribing. A third part of the study consisting of a health economic analysis is underway.

We chose a cluster RCT design (randomized practices) as the impact of intervention might bring about practice pattern changes. In the component of the study looking at patient outcomes we planned an individual-level analysis of depressive symptoms, accounting for the clustering of patients within practices, since this seemed more clinically salient than practice-level outcomes in this part of the analysis. An implication of this decision, however, is that the full benefits of randomization are lost since the unit of analysis differs from the unit of randomization.

## Methods

### Aim, study design and setting

The aim of this study was to evaluate whether training family physicians (FPs) in the AMHPSP would lead to greater improvements in patients’ depression scores (primary outcome) compared to the control group.

This was a multicentre two-parallel group, controlled trial in which practices were randomly allocated to the intervention arm (INT) or control arm and allocation was masked in the outcome assessment. Physicians in practices assigned to the intervention group attended the AMHPSP training whereas physicians assigned to the control group continued with treatment as usual (TAU).

### Participant eligibility

#### Physician practices

Seventy-seven NS community-based family practices identified through associations, presentations, and promotional letters. Interested physicians provided written informed consent and enrolled between October 2013 and January 2014 (111 FP). Physicians actively seeing patients remunerated by any method (fee-for-service/alternative funding program etc.) with a NS practice license were eligible to participate.

Eligible patients were identified by their physician. Inclusion criteria included over 18 years of age, diagnosis of depression, PHQ-9 score ≥ 10**,** ability to read/speak English (grade 6 level)**,** intact cognitive functioning (physician judgment). Exclusion criteria included active treatment with antidepressants within 5 weeks and psychotherapy within 3 months of enrollment, urgent/emergent medical or psychiatric condition (physician judgment). Patients were enrolled between June 2014 and May 2015, with the last follow-up visit in November 2015.

### Procedure

#### Practice recruitment, randomization and masking

Seventy-seven practices (111 community-based FP) were enrolled. Practice allocation was concealed at cluster level through a unique practice number (1–77) assigned by the principal Investigator (PI): B.L-H. Practices were stratified on number of physicians/practice, as well as urban and rural setting before randomization. An investigator (SP) not involved in trial operations assigned the randomization sequence. STATA, version 12 (College Station, TX, 2012) was used to generate the randomization sequence from a binomial distribution—probability of success of 0.5.

Patients and physicians’—assigned unique identifier codes—were linked to their corresponding practice number. The PI kept a master file containing participant names with ID codes in a secure location. Study databases contained de-identified information. We could not conceal arm allocation from physicians for obvious reasons. However, the RC (data collector) and outcome assessors (independent researchers) were blinded to group assignment. In addition—although we could not guarantee patient blinding—physicians were asked not to disclose group allocation to their patients.

**Fig. 1:**
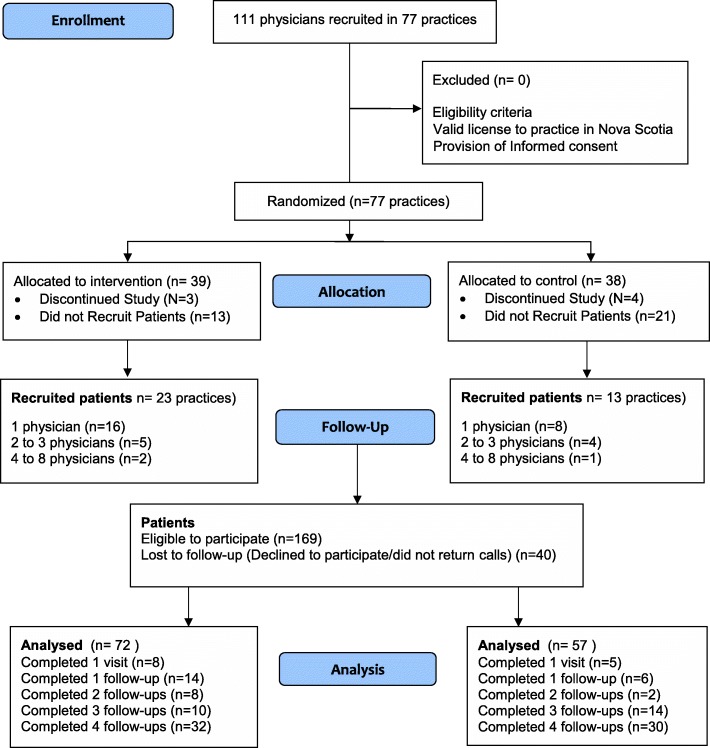
Participant Flow Diagram: Allocations, Attrition

Practices ranged from 1 to 6 physicians (INT), and 1–8 physicians (Control). Thirty-nine practices were allocated to the intervention group, and 38 to the control group. Nine percent of targeted practices withdrew prior to intervention delivery. The main reason; lack of time for study specific requirements. Only 23 of 36 (64%) of intervention and 13 of 34 (38%) of control practices recruited patients for the same reason (Fig. 1). Physician baseline characteristics such as age, gender, years of practice, pattern of work (full-time, part-time etc.), practice size, number of unique annual patients were collected.

#### Intervention

Between February–June 2014, physicians in practices assigned to the intervention group attended three half-day interactive workshops delivered by the BC team, the Adult Mental Health Practice Support Program (AMHPSP). The AMHPSP consists of three half-day interactive workshops delivered by the BC team, interspaced by 5–6 week action periods during which physicians practiced what they learned. The program introduces an organized approach that takes learners through a problem and strength-based assessment to the development of an action plan. Key components included 3 supported self-management strategies for mild to moderate depression and anxiety meant to help shift responsibility from primary care provider to a shared responsibility with the patients engaging them in illness management and their recovery; the Cognitive Behavioral Interpersonal Skills workbook incorporating a comprehensive mental illness screening tool and patient user friendly supported self-management handouts based on evidence-based cognitive behavioural therapy principles; the Canadian Mental Health Association (CMHA) Bounce Back program, a telephone guided CBT-based service; and an Antidepressant Skills Workbook [59]. Contact-based education occurred through first-voice advocates sharing their journey [60]. Office staff members attended the training and Mental Health First Aid [61] to increase their comfort caring for patients. Local psychiatrists and allied health professionals (counsellors, psychologists, clinicians etc.) attended training solely to familiarize themselves with the program. During action periods, a coordinator offered on-site guidance on practical office redesign to enhance the implementation of new learnings and tools, and shares experiences, challenges, and recommendations with stakeholders. Physicians had flexibility to use tools and strategies as clinically judged. Intervention group FPs received a learning stipend (CAN$3274.20) to participate in workshops and action periods but remunerated through usual methods to manage their patients. The control group practices received the training November 2015 (at study end) including the learning stipend.

#### Patient recruitment

Once the intervention group completed the training (June 2014), all FPs from both intervention and control groups were asked to identify 3–4 consecutive patients with a clinical diagnosis of depression, and a Patient Health Questionnaire PHQ-9 score ≥ 10. To minimize damaging the doctor/patient relationship (patients refusing or feeling-pressured to participate [62]), willing patients’ were contacted by the research coordinator (RC) who described the study, administered and obtained written consent, and assessed study eligibility. Eligible patients were assigned to the same arm as their physician. Once enrolled, patients’ own FP continued to manage the depression. A total of 169 patients were eligible to participate. Forty of 169 (24%) declined or did not return calls to complete baseline data. We enrolled 129 patients (intervention *n* = 72, control *n* = 57) all included in the analysis (Fig. 1).

#### Outcome measures

For depression scores (primary outcome), we used the PHQ-9. The PHQ-9 covers the nine DSM-5 symptom-based criteria for major depressive disorder. Total score range: 0–27; 0–4 not depressed; 5–9 mild; 10–14 moderate; 15–19 moderate-severe; 20–27 severe [63–66]. (Score ≥ 10; sensitivity 88%, specificity; 88%; positive likelihood ratio, 7.1). During scale development, criterion validity was assessed against the Structured Clinical Interview (SCID) [66, 67].

Occupational/general functioning (secondary outcomes) were assessed with the Lam Employment Absence and Productivity Scale (LEAPS) and the Sheehan Disability Scale (SDS) respectively. The LEAPS⎯a 7-item scale⎯assesses workplace impact of major depression [68]. Total score range = 0–28; 0–5 = none-minimal impairment; 6–10 = mild; 11–16 = moderate; 17–22 = severe; 23–28 = very severe. The SDS rates disruption (0–10) in each domain⎯work/school; social/leisure activities; family life/home responsibilities, (Total score range = 0–30; lower scores signify less disruption) [69].

Physician frequency of antidepressant prescribing, patient satisfaction, and quality of life (exploratory outcomes), were assessed with the Client Service Receipt Inventory, an extensively validated inventory, [70] the Client Satisfaction Inventory (CSI), and the Medical Outcomes Study Short-Form (SF-36) respectively [71–73]. Outcome measures were collected at baseline, 1, 2, 3, 6 months. All scales are validated as patient self-report, as well as telephone-collection methods. Patients could complete questionnaires over the phone, in writing or digitally online.

#### Early termination

The study was prematurely terminated in May 2015 for the following reasons. Patient recruitment completely stopped within 6 months of the enrollment start-date due to concomitant primary care health-care transformation by health-system leaders. This resulted in physicians’ reports of requiring more time for each type of appointment in order to effectively provide quality comprehensive care within in-office time constraints, thus reducing available time they could allocate to the study. The research team’s substantial efforts to re-engage physicians through evidenced-based recruitment “Best-Practices” had minimal impact. We therefore choose to report results and our challenges as useful information for future study designs, and as an exploratory assessment of the intervention.

#### Ethics statement

The NS Multisite and University of Calgary Research Ethics Boards approved this study, registration www.clinicaltrials.gov #NCT01975948.

### Statistical methods

#### Sample size

We calculated a sample size of 100 evaluable patients/arm to achieve 80% power to detect between-group differences in PHQ-9 mean of 2 points, with a significance level (α) of .05, 2-sided test, and assumed standard deviation of 5 points. A compensatory increase to 166 patients/arm was needed using a design effect of 1.1 (intra cluster correlation of 0.05 for patient outcomes based on a study by Murphey et al., [74] and 3 patients/practice on average), and the assumption that 66% of patients would provide adequate follow-up data, accounting for attrition.

### Analysis

Between-group mean differences of PHQ-9 scores during follow-up were assessed as a group-by-time interaction. In order to take advantage of the multiple measures of data, we used a multi-level mixed-model analysis⎯with patients clustered within practices and PHQ-9 ratings clustered within patients. As the pattern of improvement over time was not expected to be linear, time points were represented in the model using indicator variables. The baseline assessment was not included in the outcome assessment except as a covariate (even though it also included a PHQ-9 rating) since these ratings occurred prior to the intervention. We conducted a modified intention to treat analysis, including all respondents who provided at least one follow-up rating. The effect of the intervention was measured as series of 3 study group by time interactions, using a likelihood ratio (LR) test to test the significance of the treatment by time interaction terms. This test produced the *p*-value used to assess the statistical significance of observed differences. Occupational, general functioning, quality of life, and client satisfaction were analyzed using the same model. Antidepressant use at any time during the 6 month study period was treated as a binary variable (Y/N). Data was analysed (SP, SK) using STATA, version 14 (College Station, TX, 2015).

Since attrition occurred during follow-up, we conducted a sensitivity analysis using last observation carried forward imputation of post-attrition PHQ-9 ratings and also conducted an analysis of completers only.

## Results

### Practice characteristics

One between-group difference was observed. Physicians assigned to intervention group were more likely to work in small practices due to one large practice being randomly assigned to the control group. Twenty-four of 36 practices (67%) who recruited patients were individual-practice FPs (Table 1).

**Table 1:** Practice Characteristics

				Patient recruitment by practices size		
	Intervention (*n* = 23)	Control (*n* = 13)	p	Yes (*n* = 36)	No (*n* = 41)	P
Practice size						
1 physician	16	8		24	37	0.11
2 to 3 physicians	5	4	0.647	9	3	
4 to 8 physicians	2	1		3	1	
Urban Setting						
	14	9	0.727	23	29	0.627
Percentage	39%	35%		64%	71%	
# patients recruited/ practice						
1 to 2	11	4		–	–	–
3 to 4	7	5	0.252	–	–	–
5 or more	5	4		–	–	–
# patients with complete f/u data						
1 to 2	13	6		–	–	–
3 to 4	6	3	0.538	–	–	–
5 or more	4	4		–	–	–

### Patient characteristics

Patients were predominantly female, employed, and post-secondary educated. There were no clinically meaningful between-group differences, except that a higher proportion of control group participants were employed as compared to intervention group participants (Table 2). Also, participants with complete follow-up data tended to be in smaller practices, had somewhat lower baseline PHQ-9 scores, and were more likely to identify as married/common-law.

**Table 2:** Patient Baseline Characteristics: Intervention group, control group, completed all follow-up time points versus those who missed one or more follow-up time points

Patient Characteristics	Intervention Group	Control Group	Completed all Follow-Up		P^
	*n* = 72	*n* = 57*	Yes (*n* = 62)	No (*n* = 67)**	
Gender					
Female	49 (68.1%)	43 (76.8%)	44 (71.0%)	48 (72.7%)	.33, .85
Marital status					
Married/common-law	38 (52.8%)	37 (66.1%)	42 (67.7%)	33 (50.0%)	.15, .05
Not married/single/divorced/ separated/widowed	34 (47.2%)	19 (33.9%)	20 (32.3%)	33 (50.0%)	
Age					
18–39	31 (43.1%)	31 (55.4%)	26 (41.9%)	36 (54.5%)	
40–59	26 (36.1%)	18 (32.1%)	26 (41.9%)	18 (27.3%)	.33, .20
60+	15 (20.8%)	7 (12.5%)	10 (16.1%)	12 (18.2%)	
Employment status					
Employed (full or part time)	38 (52.8%)	43 (78.2%)	43 (69.4%)	38 (58.5%)	
Unemployed	9 (12.5%)	3 (5.5%)	4 (6.5%)	8(12.3%)	.01, .37
Retired/student/at-home/other	25 (34.7%)	9 (16.4%)	15 (24.2%)	19 (29.2%)	
Education					
Some elementary or High school	9 (12.5%)	3 (5.4%)	5 (8.1%)	7 (10.6%)	
High school/some post-secondary	23(31.9%)	21 (37.5%)	18 (29.0%)	26 (39.4%)	.40, .32
Post-secondary diploma/degree +	40 (55.6%)	32 (75.0%)	39 (62.9%)	33 (50.0%)	
Mother tongue					
English	66 (91.7%)	55 (98.2%)	59 (95.2%)	62 (93.9%)	
Other	6 (8.3%)	1 (1.8%)	3 (4.8%)	4 (6.1%)	.14, 1.0

*One respondent did not provide demographic data. Percentages based on *n* = 56. Two respondents did not provide employment status data

**One respondent did not provide demographic data. Percentages based on *n* = 66. Two respondents did not provide employment status data

^Fisher’s exact test was used

First *p* value pertains to INT/control comparison, the second for completed vs not comparison

### Primary outcome

**Fig. 2:**
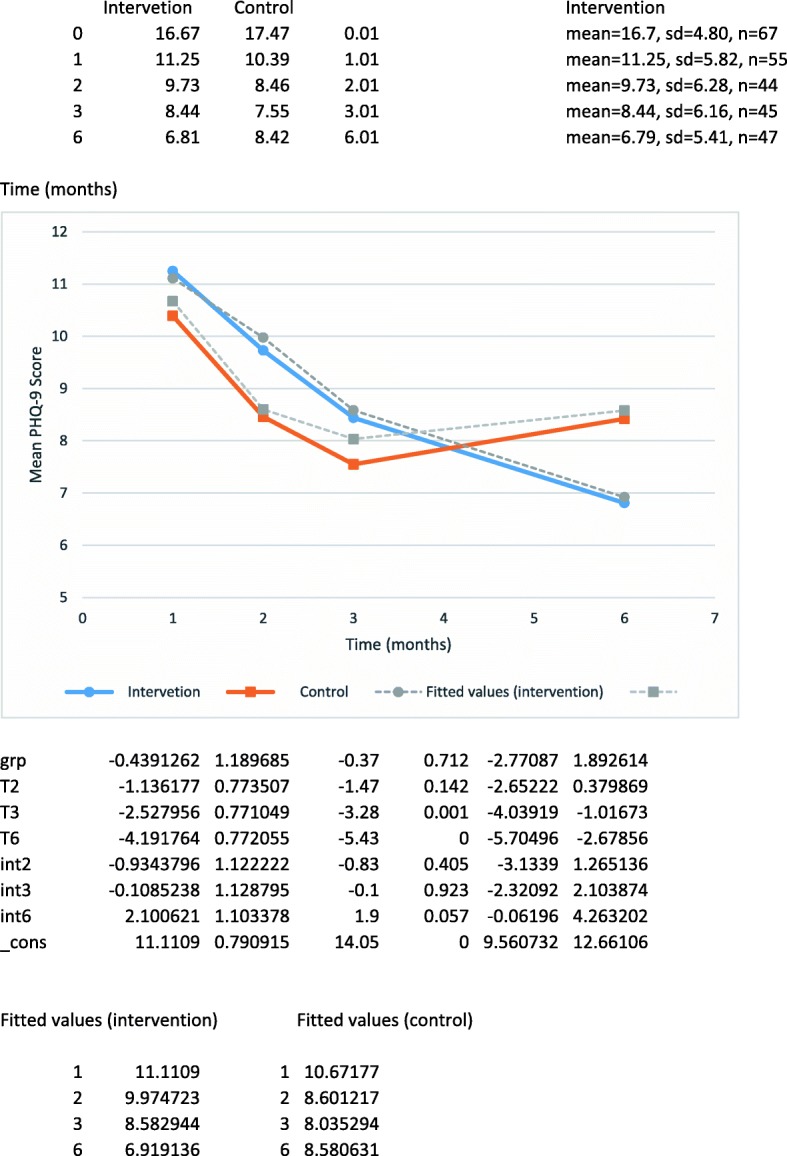
Between-group mean differences of PHQ-9 scores, group-by-time interaction

PHQ-9 scores diminished in both groups (Fig. 2). The treatment group, however, had a progressive diminishment in mean PHQ-9 scores, whereas the control group did not continue to decrease after the 3-month follow-up visit. The multi-level mixed model results were reflective of a significant treatment effect (LR for treatment group by time interactions, chi2(3) = 7.96, *p* = .047), which was essentially unchanged after adjusting for baseline depressive symptoms (LR Chi2(3) = 9.04, *p* = 0.029). Addition of age categories and sex to the model (the age categories were depicted in the dataset using indicator variables representing six age groups) did not change the result (LR for treatment group by time interactions terms, chi2(3) =10.40, *p* = 0.006). Consistent with the pattern seen in Fig. 2, which suggests that differences were only evident in the final time interval, removal of the first 2 time-by-treatment group interactions did not significantly affect the fit of the models, e.g. for the model with adjustments for age, sex and baseline depressive symptoms (LR comparing the original to the reduced model, chi2(1) = 1.07, *p* = 0.301), indicating no significant loss of fit with removal of the two interaction terms.

Because of the imbalance in employment observed in Table 2, the analysis was repeated including an indicator term for full time employment status. The results were unchanged: LR chi2(3) = 10.37, Prob > chi2 = 0.0157.

### Secondary outcomes

Between-group changes on LEAPS and SDS, SF-36, and CSI were not significantly different (See Additional file 1).

### Exploratory outcomes

Antidepressant use at any time during the 6 months study period was significantly lower in the intervention group compared to the control group Fisher’s exact test, *p* = 0.003. (Control group = 68.4%; Intervention group = 41.7%). In a sensitivity analysis using LOCF imputation there was no evidence of a treatment effect as assessed by the three group by time interactions (LR for the treatment group by time interactions, chi2(3) = 5.28, Prob > chi2 = 0.153). However, in the completer analysis, the effect remained significant: LR for the treatment group by time interactions chi2(3) = 7.96, Prob > chi2 = 0.047. Fifty-one percent of INT patients used program tools/strategies. The top 3 were CBT-based with BounceBack being highest (51% of patients who used tools, 26% of INT patients) (Table 3).

**Table 3:** Antidepressant Prescribing at 6 months: Intervention and control groups Strategies used: Intervention

Antidepressant Use (%)	INT			CONTROL
	41.7%			68.4%
Specific tools used	N	% of participants (*n* = 37)	% of all INT participants (*n* = 72)	
Referral to BB	19	51.4%	26.4%	0
ASW resources	11	29.7%	15.3%	0
CBIS resources	9	24.3%	12.5%	0
BB DVD provided	6	16.2%	8.3%	0
Physician telephone follow-up	3	8.1%	4.2%	0
DAI administered	3	8.1%	4.2%	0
Total*	51	116%	71%	0

*patients used 1 or more tools

*Mentioned tools: Diagnostic Assessment Interview (DAI) administered; Antidepressant Skills Workbook (ASW) provided; Referral to Bounce Back (BB); Bounce Back DVD provided; Cognitive Behavioural Interpersonal Skills (CBIS) resources provided; physician follow-up by telephone

## Discussion

Results of this study provide tentative evidence of the AMHPSP’s effectiveness in improving patient depression scores. A large majority of INT patients (71%) reported using the program tools and strategies, the most frequently used was the BounceBack program (Table 3). Anecdotal FP reports suggested that BounceBack program offers increased support by shared patient management. PHQ-9 scores diminished in both groups from baseline to 3 months due to the episodic nature of depression expected to cause regression to the mean, and possibly as a result of placebo responders through repeated encounters with the research data collector. In psychiatric disorders such as depression, this non-specific treatment effect is a well-recognized component in all psychotherapeutic encounters [75]. The treatment group, however, had a progressive diminishment in mean PHQ-9 scores, whereas the control group did not continue to decrease after the 3-month follow-up visit (Fig. 1).

Further research is needed in this area as our study did not achieve its pre-planned sample size, lending to the possibility of Type II error. Also, the effect observed was small only evident at a single time interval, between 3 and 6 months, a finding that may have occurred as a result of Type I error. There was substantial attrition, and LOCF imputation did not result in preservation of the effect. However, the pattern of between-group change in mild/moderate depression scores over time is suggestive of a possible treatment effect. Furthermore, these changes occurred despite reduced prescribing of medication by FPs. (Table 3) Participants with complete follow-up data had somewhat lower baseline PHQ-9 scores, which is consistent with the AMHPSP’s target population: mild to moderate depression, hence future studies should consider including only this group. Future studies should also strongly consider using a practice-level unit of analysis since randomization in such trials must by necessity occur at the practice level.

In a context where collaborative care has proven difficult, (e.g., where primary care is delivered in independent practice settings and/or no funding mechanisms for collaborative care arrangements, we were looking at ways of implementing time-efficient evidence-based strategies to increase physicians’ comfort and skills, decreasing anxiety/avoidance treating highly prevalent depression conditions. We do not feel there are or will ever be enough mental health physicians/clinicians to support FPs especially in rural and remote areas. Therefore, responsibility to care for the mentally ill largely rests with family physicians. We hear time and again that primary care knowledge gaps contribute to unrecognized and undertreated mental illness. However, training efforts aimed to improve mental health management and patient outcomes remain questionable [30]. Training efforts need to include time efficient tools FPs can feasibly implement in the realistic context of a busy practice.

The AMHPSP cannot address all barriers, however it is novel recognizing that FPs need in-practice support that expand well beyond a simple education program or workshop. It includes many theoretically crucial ingredients required to fill knowledge gaps, through organized training that creates a substantial sustainable change, in the delivery of patient-centered respectful care, by FPs that fits their busy schedule, targets stigma, and supports physicians and patients through incentives, resources, and tools engaging patients in recovery efforts. Most patients want to participate in their recovery. Where better to learn the skills than in a trusted, safe place with their physician, even in settings where access to specialty care is difficult or unavailable?

To our knowledge, this study is the first RCT of its kind specifically evaluating a practice support program focusing on mental health as it is meant to be used in a real-world environment. We believe this provides insight on the potential impact of the AMHPSP training on patients’ clinical outcomes and practical applicability of delivering the program in primary care. Furthermore improvement in depression scores occurred despite reduced prescribing of medication by FPs which suggests possible cost offsets due to reduced prescribing costs (Table 3). Also, prior analyses indicated reductions in social distance preferences and increased confidence and comfort with the training, beneficial effects not captured by the analysis of patient-reported outcomes presented here^58^.

## Limitations

First, this was a controlled trial, but randomization occurred at the practice level rather than individual patients. Because the analysis occurred at the level of patients, the benefits of randomization (equal distribution of confounding variables) cannot be assumed to have fully manifested in the analysis. Indeed, as some practices failed to recruit patients at all, the patient data analysis may have been influenced by selection bias despite the randomization. Whereas the random assignment would help ensure that the practices assigned to each group had similar characteristics, including unmeasured ones, it does not ensure that the ones that recruited patients were comparable on such characteristics. The significant difference in PHQ-9 scores was observed later in follow-up, at which point some physician and patient attrition had occurred, and this attrition may have caused bias. The lack of significant effects in a sensitivity analysis using LOCF imputation heightens these concerns. In addition, the there was an imbalance in patient group sizes (intervention *n* = 72, Control *n* = 57) which could have further affected the specific effects of the intervention compared to TAU (Fig. 1). However, the mixed model analysis methods permits the use of all data (e.g. data points from earlier in follow-up even when lost to follow-up), and may help to control bias that may arise due to attrition.

As noted above, future studies randomizing such interventions by practice should consider the use of practice-level outcome measures that would allow the analysis to derive full benefit from the randomization, a larger sample size and employ effective strategies to prevent attrition, especially since the benefits of this type of intervention may unfold over a time frame of several months. Third, physician participants were volunteers receiving a learning stipend, therefore may not be representative of FPs in general, may have a special interest in the subject matter. Although some aspects of the study could not be blinded, performance bias was minimized by data collector and outcome assessors blinding as well as the use of widely validated scales⎯more specifically the PHQ-9 for our primary outcome. We took measures also to minimize the risk of patient selection bias by asking physicians to identify 3 consecutive patients with a diagnosis of depression.

Finally, a longer patient follow-up period may be important to shed light if divergence of depression scores would further increase, or reveal a significant difference in quality of life/occupational functioning as these outcomes may take longer to improve. Since the study sample consisted mostly of white, married, middle-age females, there should be caution in generalizing results beyond this group. However, the approach is applicable to all prepaid health insurance plans in the US and to Canada’s single payer system [42].

In assessing satisfaction, most patients reported high baseline physician satisfaction⎯ceiling effect. An outcome measure specific to mental healthcare would have been a better choice.

## Conclusion

This study provides preliminary evidence that well-designed novel skills-based PSPs that promote integration of mental health into primary care may contribute to mental health strategies in improving mental health care. It also highlights the difficulties in evaluating community-delivered interventions.

## Additional files

Additional file 1: Tables: Mean, standard deviation (SD) and number of non-missing observations. (PDF 246 kb)

Additional file 2: Data access document. (DOCX 12 kb)

## Abbreviations

AMHPSP: Adult mental health practice support program
BC: British Columbia
CBT: cognitive behaviour therapy
CMHA: Canadian mental health association
CSI: Client satisfaction inventory
FP: Family physician
LEAPS: Lam employment absence and productivity scale
LOCF: last observation carried forward
LR: likelihood ratio t
NS: Nova Scotia
PHQ-9: Patient health questionnaire-9
PI: Principal investigator
RC: Research coordinator
SCID: Structured clinical interview
SDS: Sheehan disability scale
SF-36: Medical outcomes study short-form
TAU: Treatment as usual
