# Supplementary material for: Controlled trial of the impact of a BC adult mental health practice support program (AMHPSP) on primary health care professionals’ management of depression
Source: BMC Fam Pract. 2018 Nov 28;19:183. doi: 10.1186/s12875-018-0862-y (PMC6262957; doi:10.1186/s12875-018-0862-y)
Supplement: Supplementary file 1 — Tables: Mean, standard deviation (SD) and number of non-missing observations. (PDF 246 kb) [file 12875_2018_862_MOESM1_ESM.pdf]

Supplementary Tables – Mean, standard deviation (sd) and number of non-missing observations.

A. Primary Analysis

**PHQ-9 Score**			Intervention	
Time (months)	#practices	mean	sd	n
0	23	16.76	4.80	67
1	20	11.25	5.82	55
2	20	9.73	6.28	44
3	17	8.44	6.16	45
6	18	6.79	5.42	47
			Control	
Time (months)	#practices	mean	sd	n
0	13	17.51	4.16	55
1	12	10.39	6.49	44
2	13	8.46	6.47	41
3	12	7.55	6.13	38
6	12	8.42	6.57	45

B. Secondary Analyses

**LEAPS Score**		Intervention	
Time (months)	mean	sd	n
0	12.75	6.59	42
1	7.13	5.96	30
2	7.16	6.88	28
3	5.96	5.06	26
6	6.03	6.12	37
		Control	
Time (months)	mean	sd	n
0	11.32	5.71	32
1	7.67	5.36	30
2	7.45	6.21	22
3	5.54	4.37	24
6	6.04	5.79	26
**SDS Score**		Intervention	
Time (months)	mean	sd	n
0	19.69	6.67	54
1	13.02	9.00	43
2	9.43	8.95	38
3	8.28	7.29	36
6	7.86	8.46	39
		Control	
Time (months)	mean	sd	n
0	19.28	7.06	68
1	13.87	8.20	52
2	13.74	9.12	39
3	10.42	9.34	43
6	7.60	7.27	43

Continued on next page…

**CSI Score***		Intervention	
Time (months)	mean	sd	n
0	152.16	22.11	50
1	152.26	23.68	40
2	155.24	23.75	35
3	153.72	22.79	33
6	150.34	25.11	39
		Control	
Time (months)	mean	sd	n
0	156.09	13.82	60
1	153.00	19.67	55
2	154.96	19.07	41
3	151.95	23.29	47
6	151.70	19.64	44
**SF36 Score****		Intervention	
Time (months)	mean	sd	n
0	53.01	14.83	54
1	62.33	15.30	44
2	67.62	15.59	39
3	67.00	18.83	39
6	69.03	21.96	46
		Control	
Time (months)	mean	sd	n
0	54.18	13.67	68
1	57.73	17.09	56
2	61.77	18.38	44
3	62.45	21.76	47
6	67.10	20.88	46

*Total scores can range from 25 to 175. Higher scores indicate greater overall satisfaction.

**Total scores can range from 0-100. Higher scores indicate higher overall functioning.
