# Supplementary material for: Controlled trial of the impact of a BC adult mental health practice support program (AMHPSP) on primary health care professionals’ management of depression
Source: BMC Fam Pract. 2018 Nov 28;19:183. doi: 10.1186/s12875-018-0862-y (PMC6262957; doi:10.1186/s12875-018-0862-y)
Supplement: Supplementary file 2 — Data access document. (DOCX 12 kb) [file 12875_2018_862_MOESM2_ESM.docx]

The data are archived on the University of Calgary Web Server at:

<http://people.ucalgary.ca/~patten/Datasets/deidentified_dataset.dta>

The data can be accessed from within Stata using the following command:

use <http://people.ucalgary.ca/~patten/Datasets/deidentified_dataset.dta>

The LOCF and completer datasets are available here:

use <http://people.ucalgary.ca/~patten/Datasets/deidentified_data_locf.dta>

use <http://people.ucalgary.ca/~patten/Datasets/deidentified_data_completer.dta>

These are the commands used to generate the p-values reported in the analysis:

tabulate TIME, summarize(PHQ_TOT)

bysort grp: tabulate TIME, summarize(PHQ_TOT)

//note that T2, T3, T6 are indicator variables for time points, in “int” terms are group x time interactions

xtmixed PHQ_TOT grp T2 T3 T6 int2 int3 int6 || PRACTICE: || idno:

est store A

xtmixed PHQ_TOT grp T2 T3 T6 || PRACTICE: || idno:

lrtest A

//repeating model with the adjustment for baseline PHQ-9. Tb is the PHQ9 score at baseline.

xtmixed PHQ_TOT grp Tb T2 T3 T6 int2 int3 int6 || PRACTICE: || idno:

est store B

xtmixed PHQ_TOT grp Tb T2 T3 T6 || PRACTICE: || idno:

lrtest B

//repeating it with age and sex also. As age groups were used, the age variable is factored. CSRI2 //represents sex

xtmixed PHQ_TOT CSRI2 i.ageR grp Tb T2 T3 T6 int2 int3 int6 || PRACTICE: || idno:

est store C

xtmixed PHQ_TOT CSRI2 I.ageR grp Tb T2 T3 T6 || PRACTICE: || idno:

lrtest C

//repeating it with only a single interaction term

xtmixed PHQ_TOT CSRI2 i.ageR grp Tb T2 T3 T6 int6 || PRACTICE: || idno:

lrtest C

//a model including an indicator for employment status.

xtmixed PHQ_TOT CSRI2 i.ageR employment grp Tb T2 T3 T6 int2 int3 int6 || PRACTICE: || idno:

est store D

xtmixed PHQ_TOT CSRI2 i.ageR employment grp Tb T2 T3 T6 || PRACTICE: || idno:

lrtest D

This is the command to compare antidepressant use (at any time during study period) between treatment and intervention groups. The antidepressant data is not in the LOCF or completer datasets.

//Antidepressant use is classified for all subjects as yes/no, the tabulation is done at a single time point. The first command shows the frequencies, the second performs the Fisher’s exact tes.

bysort grp: tab antidep if TIME==6

tab antidep grp if TIME==6, exact
